# Relationship between morphological features and kinetic patterns of enhancement of the dynamic breast magnetic resonance imaging and clinico-pathological and biological factors in invasive breast cancer

**DOI:** 10.1186/1471-2407-10-8

**Published:** 2010-01-08

**Authors:** Oscar Fernández-Guinea, Alejandro Andicoechea, Luis O González, Salomé González-Reyes, Antonio M Merino, Luis C Hernández, Alfonso López-Muñiz, Paz García-Pravia, Francisco J Vizoso

**Affiliations:** 1 Unidad de Investigación, Hospital de Jove, Gijón-Spain; 2 Servicio de Radiodiagnóstico, Hospital de Jove, Gijón-Spain; 3 Servicio de Cirugía General, Hospital de Jove, Gijón-Spain; 4 Servicio de Anatomía Patológica, Hospital de Jove, Gijón-Spain; 5 Instituto Universitario de Oncología del Principado de Asturias, Oviedo-Spain; 6 Servicio de Anatomía Patológica, Hospital de Cabueñes, Gijón-Spain; 7 Departamento de Morfología y Biología Celular, Universidad de Oviedo-Spain

## Abstract

**Background:**

To investigate the relationship between the magnetic resonance imaging (MRI) features of breast cancer and its clinicopathological and biological factors.

**Methods:**

Dynamic MRI parameters of 68 invasive breast carcinomas were investigated. We also analyzed microvessel density (MVD), estrogen and progesterone receptor status, and expression of p53, HER2, ki67, VEGFR-1 and 2.

**Results:**

Homogeneous enhancement was significantly associated with smaller tumor size (T1: < 2 cm) (p = 0.015). Tumors with irregular or spiculated margins had a significantly higher MVD than tumors with smooth margins (p = 0.038). Tumors showing a maximum enhancement peak at two minutes, or longer, after injecting the contrast, had a significantly higher MVD count than those which reached this point sooner (p = 0.012). The percentage of tumors with vascular invasion or high mitotic index was significantly higher among those showing a low percentage (≤ 150%) of maximum enhancement before two minutes than among those ones showing a high percentage (>150%) of enhancement rate (p = 0.016 and p = 0.03, respectively). However, there was a significant and positive association between the mitotic index and the peak of maximum intensity (p = 0.036). Peritumor inflammation was significantly associated with washout curve type III (p = 0.042).

**Conclusions:**

Variations in the early phase of dynamic MRI seem to be associated with parameters indicatives of tumor aggressiveness in breast cancer.

## Background

Magnetic resonance imaging (MRI) plays an important role in the evaluation of the extent of breast cancer by revealing multifocal tumor growth in patients who are candidates for conservative breast surgery [1]. MRI permits us to explorer two concepts: First, we are able to analyze the morphologic characteristics of the lesions with high spatial resolution, such as the margin morphology (smooth, irregular or spiculated) or the internal architecture of the tumors (represented as internal mass enhancement: homogeneous, heterogeneous or rim enhancement) [1-3]. Second, we can also obtain dynamic data derived from the kinetic patterns of lesion enhancement after the administration of contrast material [4]. These latter MRI parameters include the behaviour of the signal intensity in the early phase after the administration of contrast material, as well as in the late postcontrast period. Likewise, this time course may be visualized in two and three-dimensional dynamic MRI series. These time-signal intensity curves allow us to determine whether the signal intensity continues to increase after the initial upstroke, cuts off and reaches a plateau, or if it just washes out. It has been demonstrated that this latter curve type is a strong indicator of malignancy, being independent of other criteria [5].

More recently, MRI appears to have an important value in estimating other aspects of interest in breast cancer, such as the assessment of axillary lymph node metastasis, or the prediction of the clinicopathological response to primary chemotherapy [3,6,7]. It has also been suggested that MRI might be useful in predicting the disease-free survival in breast cancer patients [8]. In addition, there are studies indicating that dynamic contrast-enhanced MRI help to predict prognostic factors and biological activity of breast cancer by revealing morphological features and enhancement parameters of the primary tumors, such as angiogenesis, degree of fibrosis [9], histological grade [10,11], negative expression of estrogen repeptor and progesterone receptor [11], vascular endothelial growth factor (VEGF) expression [9] or HER-2 overexpression [12]. In this context, the objectives of this study were to investigate the relationship between the MRI features of breast cancer and some other of their clinicopathological and biological characteristics, such as vascular invasion, peritumoral inflammation or VEGF-receptor-1 and 2.

## Methods

### Patient selection and characteristics

This study comprised 68 women consecutively diagnosed of early invasive breast cancer (without distant metastasis at time of initial diagnoses) and treated between 1999 and 2006. Initially, the lesions were detected by physical examination, mammography, or ultrasonography. All of the women did not receive any type of neoadjuvant therapy. In addition, for the 68 cases we obtained sufficient tissue in the paraffin blocks used for manufacturing the tissue arrays (TAs). The patients' age ranged from 30 to 83 years (mean, 55.5 years), and the tumor size between 0.6 cm and 12 cm (mean, 2.3 cm). A total of 54 tumors were of the ductal type, 9 of the lobular type, 2 mucinous, one medullar, one tubullar and one papilla. Other patient characteristics evaluated in this study are listed in Table 1. The histologic grade was assessed according to criteria reported by the Nottingham modification of Bloom and Richardson score (SBR) [13]. DCIS component was present in 16 cases (23.5%)

**Table 1:** Relationship between morphological features, kinetic patterns of enhancement and clinicopathological characteristics of patients

		Morphological features				N°	Kinetic features						
				
		Margin		Enhancement internal			Peak of maximum enhancement		% of maximun enhancement < 2'		Curve Type		
				
Characteristics	N°*	A	B	Hom	Het/Rim		< 2'	≥ 2'	≤ 150%	> 150%	I	II	III
**Total cases**	64					68							

**Menopausal status**													

Premenopausal	26	4 (15.4)	22 (84.6)	4 (15.4)	22 (84.6)	29	5 (17.2)	24 (82.8)	14 (48.3)	15 (51.7)	2 (6.9)	11 (37.9)	16 (55.2)

Postmenopausal	38	12 (31.6)	26 (68.4)	8 (21.1)	30 (78.9)	39	12 (30.8)	27 (69.2)	25 (64.1)	14 (35.9)	3 (7.7)	13 (33.3)	23 (59)

**Tumor size**				**p = 0.015**									

T1 (<2 cm)	42	14 (33.3)	28 (66.7)	**12 (28.6)**	**30 (71.4)**	43	10 (23.2)	33 (76.8)	28 (65.1)	15 (34.9)	4 (9.3)	13 (30.2)	26 (60.5)

≥ T2 (≥ 2 cm)	22	2 (9.1)	20 (90.9)	**0**	**22 (100)**	25	7 (28)	18 (72)	11 (44)	14 (56)	1 (4)	11 (44)	13 (52)

**Nodal status**													

N (-)	35	9 (25.7)	26 (74.3)	7 (20)	28 (80)	35	10 (28.6)	25 (71.4)	20 (57.1)	15 (42.9)	3 (8.6)	10 (28.6)	22 (62.8)

N (+)	29	8 (27.6)	21 (72.4)	6 (20.7)	23 (79.3)	33	8 (24.2)	25 (75.8)	19 (57.6)	14 (42.4)	2 (6.1)	15 (45.4)	16 (48.5)

**Histological grade**													

I	22	6 (27.3)	16 (72.7)	5 (22.7)	17 (77.3)	24	4 (16.7)	20 (83.3)	14 (58.3)	10 (41.7)	4 (16.6)	10 (41.7)	10 (41.7)

II	24	6 (25)	18 (75)	5 (20.8)	19 (79.2)	26	7 (26.9)	19 (73.1)	13 (50)	13 (50)	1 (3.8)	8 (30.8)	17 (65.4)

III	18	4 (22.2)	14 (77.8)	2 (11.1)	16 (88.9)	18	6 (33.3)	12 (66.7)	12 (66.7)	6 (33.3)	0	6 (33.3)	12 (66.7)

**Vascular invasion**									**p = 0.016**				

Yes	27	8 (29.6)	19 (70.4)	4 (14.8)	23 (85.2)	29	6 (20.7)	23 (79.3)	**22 (75.9)**	**7 (24.1)**	2 (6.9)	11 (37.9)	16 (55.2)

**No**	37	8 (21.6)	29 (78.4)	8 (21.6)	29 (78.4)	39	11 (28.2)	28 (71.8)	**17 (43.6)**	**22 (56.4)**	3 (7.7)	13 (33.3)	23 (59)

**Mitotic Index**							**p = 0.036**		**p = 0.03**				

≤ 5	29	6 (20.7)	23 (79.3)	5 (17.2)	24 (82.8)	33	**4 (12.1)**	**29 (87.9)**	**14 (42.4)**	**19 (57.6)**	4 (12.1)	13 (39.4)	16 (48.5)

>5	35	10 (28.6)	25 (71.4)	7 (20)	28 (80)	35	**13 (37.1)**	**22 (62.9)**	**25 (71.4)**	**10 (28.6)**	1 (2.9)	11 (31.4)	23 (65.7)

**Peritumor inflammation**											**p = 0.042**		

Yes	36	10 (27.8)	26 (72.2)	7 (19.4)	29 (80.6)	37	11 (29.7)	26 (70.3)	23 (62.2)	14 (37.8)	**1 (2.7)**	**10 (27)**	**26 (70.3)**

No	28	6 (21.4)	22 (78.6)	5 (17.9)	23 (82.1)	31	6 (19.4)	25 (80.6)	16 (51.6)	15 (48.4)	**4 (12.9)**	**14 (45.2)**	**13 (41.9)**

* Four cases showed a non-nodular enhancement, so the morphological features were analyzed in only 64 cases, and the kinetic features in all 68 cases. Data are expressed as number of cases (percentage).

Abbreviations: A, well-defined (regular); B, ill-defined (irregular or spiculated); Hom: homogeneous; Het: heterogeneous.

Women were treated according to the International European Guidelines. The study adhered to national regulations and was approved by our institution's Ethics and Investigation Committee.

### MR Imaging

All studied subjects had exactly the same MR sequences and parameters. MRI was performed at 1.5 Teslas (Echospeed Signa; General Electric Medicale Systems, Milwaukee, WI, USA). After the informed consent was obtained, patients were placed in the prone position and examined using standard dedicated bilateral breast coils. The imaging protocol consisted of an initial scout view that provided axial, coronal, and sagittal images of both breast. These images were used to exactly localize the spatial distribution of the breast parenchymal volume. The subsequent axial dynamic series were then positioned to cover the whole parenchyma.

Before administration of contrast material, T1-weighted frames were acquired in the axial plane (FSPGR -fast spoiled gradient echo- 3D; FA -flip angle-, 10°; TR, 9.9 milliseconds; TE, 4.2 milliseconds; NEX, 1; 2-3 mm slice thickness with no gap; 512 × 192 matrix; in-plane resolution, 0.6 × 1.8; frequency was in the anteroposterior direction). Acquisition of dynamic imaging started 10s after the intravenous injection of 0.2 mmol per kilogram body weight of gadopentetate dimeglumine (Gd-DTPA) (Magnevist; Schering, Madrid, Spain), followed by a 20 ml saline solution flush, at an injection rate of 2 mL/s, following by six series, with lasted 80s each for a total imaging time of slightly over nine minutes. The injection unit contained no magnetic components and operated with pressurized air. Imaging time with this frame was approximately 80s for a total imaging time of slightly over nine minutes. Acquisition of dynamic imaging started 10s after contrast injection, following by six frames. Each frame had 64 slices.

### Image Analysis

All images were evaluated with the Functool algorithm on the Advantage Windows Workstation (General Electric Medical Systems) by consensus between two radiologists (O.F.G. and P.G.P.), with a wide experience in breast imaging.

After the dynamic series were obtained, image subtraction was performed to suppress the fat signal, and enhancing lesions were identified on the subtracted images. To verify the presence of a contrast-enhancing lesion and to exclude subtraction artifacts, we also re-identified the lesions on the non-subtracted images.

For each suspected lesion included in the dynamic slices, the following morphologic features were recorded: site, size, margins, and type of enhancement. To evaluate kinetics, a small region of interest (ROI) is placed selectively over the most intensely enhancing area of the lesion. The ROI size was always greater than three pixels, and without upper limit. The ROI was placed in the rim enhancement during the dynamic study, when the tumors showed this finding.

The lesion margins were described as well-defined (regular) or ill-defined (irregular or spiculated). The enhancement after Gd-DTPA administration was classified as homogeneous, heterogeneous, or rim enhancement.

Quantitative analysis of Gd-DTPA uptake was based on a two-compartment model of the pharmacokinetic behaviour of contrast medium according to three parameters: wash-in rate, wash-out rate and amplitude of uptake. The enhancement rate was calculated according to the following enhancement formula: enhancement rate = [(SI_post _- SI_pre_)/SI_pre_] × 100 (%), where SI_pre _and SI_post _are the precontrast and the postcontrast signal intensities, respectively [5]. According to the most commonly accepted criteria, signal-intensity curves were classified into three categories: persistent enhancement (type I), plateau (type II) and washout (type III). Plateau and wash-out curves showed the peak of enhancement within the early phase of contrast administration. Plateau curves leveled off, whereas wash-out curve demonstrated a decrease in signal intensity after reaching a maximum signal intensity value. The following parameters of the dynamic signal before Gd-DTPA administration were also considered: peak of maximum intensity before 2 minutes, within 2 to 4 minutes (including 2 and 4 minutes), within 4 to 6 minutes (>4 minutes and 6 minutes inclusived), and after 6 minutes; percentage of maximum uptake before 2 minutes (three groups: <100%, 100%-150%, and >150%); median (range) of the maximum uptake value before 2 minutes; and the median (range) time to reach the maximum uptake.

### Tissue arrays and immunohistochemistry

Routinely fixed (overnight in 10% buffered formalin), paraffin-embedded tumor samples stored in our pathology laboratory files were used in this study. Histopathologically representative tumor areas were defined on haematoxylin and eosin-stained sections and marked on the slide. Tumor tissue array blocks were obtained by punching a tissue cylinder (core) with a diameter of 1.5 mm through a histologically representative area of each 'donor' tumor block, which was then inserted into an empty 'recipient' tissue array paraffin block using a manual tissue arrayer (Beecher Instruments, Sun Prairie, WI, USA). Areas of non-necrotic cancerous tissue from tumoral center were selected for arraying by an experienced pathologist (L.O.G.). Two cores were used for each case. From the 68 tumor samples available, two tissue array blocks were prepared, each containing more than 30 samples, as well as internal controls including four normal breast tissue samples from two healthy women that underwent reductive mammary surgery.

Serial 5-μm sections were consecutively cut with a microtome (Leica Microsystems GmbH, Wetzlar, Germany) and transferred to adhesive-coated slides. One section from each tissue array block was stained with Haematoxylyn and eosin, and these slides were then reviewed to confirm that the sample was representative of the original tumor. Immunohistochemistry was done on these sections of tissue arrays (TA) using a TechMate TM50 autostainer (Dako, Glostrup, Denmark). Sections were then incubated with the following (all from Dako): mouse anti-ER clone 1D5 at a dilution of 1/50, anti-PR clone PgR 636 at a dilution of 1/50, anti-Ki67 clone MIB-1 at a dilution of 1/50, anti-p53 clone DO-7 at a dilution of 1/75 and rabbit policlonal anti-HER-2/neu oncoprotein at a dilution of 1/250, mouse anti-CD34 (clone QBEnd/10 at dilution 1:1) (Lab Vision Corporation, Fremont CA, USA), rabbit anti Flt -1/VEGFR-1 (dilution 1:1), and Flk1/KDR/VEGFR-2 (dilution 1:1) (Lab Vision Corporation, Fremont, CA, USA); all the dilutions were made in Antibody Diluent, (Dako) for 30 min at room temperature.

Tissue sections were deparaffinized in xylene, and then rehydrated in graded concentrations of ethyl alcohol (100%, 96%, 80%, 70%, then water). To enhance antigen retrieval for some antibodies, TA sections were microwave-treated (H2800 Microwave Processor, EBSciences, East Granby, CT, USA) in citrate buffer (Target Retrieval Solution, Dako) at 99°C for 16 min. Endogenous peroxidase activity was blocked by incubating the slides in peroxidase-blocking solution (Dako) for 5 min. The EnVision Detection Kit (Dako) was used as the staining detection system. Sections were counterstained with hematoxilin, dehydrated with ethanol, and permanently coverslipped.

### TA analysis

For VEGFR-1 and 2, the location of immunoreactivity, as well as the percentage of stained cells and their intensity, were determined. All the cases were semiquantified for each protein-stained area. An image analysis system with the Olympus BX51 microscope and Soft analysis (analySIS^®^, Soft imaging system, Münster, Germany) were used as follows: tumor sections were stained with antibodies according to the method explained above and counterstained with haematoxylin. There were different optical thresholds for both stains. Each core was scanned with a 400 × power objective in two fields per core. Fields were selected by searching for the highest staining area and finally we average the staining score. The computer program selected and traced a line around antibody-stained areas (higher optical threshold: red spots), with the remaining, non-stained areas (haematoxylin-stained tissue with lower optical threshold) standing out as a blue background. Each field has an area ratio of stained (red) versus non-stained (blue). A final area ratio was obtained after averaging two fields. To evaluate immunostaining intensity we used a numeric score ranging from 0 to 3, reflecting the intensity as follows: 0, no staining; 1, weak staining; 2, moderate staining; and 3, intense staining. Using an Excel spreadsheet, the mean score was obtained by multiplying the intensity score (I) by the percentage of stained cells [14] and the results were added together (total score: I × PC). This overall score was then averaged with the number of cores that were analyzed for each patient. If there was no tumor in a particular core, then no score was given. In addition, for each tumor, the mean score of two core biopsies was calculated.

Five fields per core, corresponding to areas of higher immunostaining and without necrosis, were evaluated for CD34, with a final area of 1 mm^2^. If there was no tumor in a particular core, ten fields were evaluated in another one in order to obtain the same final area. We obtain a total score and this is the value of MVD in each tumor.

Staining for ERs and PgRs was scored according to the method described by Allred et al. [15]. p53 was assessed by the number of positively stained nuclei, with greater than 25% of stained cells indicating a positive result. The HER-2 staining was made by immunohistochemistry with rabbit polyclonal antibody from Dako and for the assessment we use the Herceptest scoring guidelines. According to that a tumor was reported 2+ when a weak to moderate complete membrane staining is observed in > 10% of tumor cells. These cases were classified as equivocal and required confirmation by FISH and a tumor was reported 3+ when a strong complete membrane staining is observed in > 10% of tumor cells. These cases were classified as positives and confirmation was not required. Ki-67 (MIB-1) was assessed by the number of positively stained nuclei, with greater than 10% of cells staining indicating a positive result. Controls included breast cancer tissue with known immunoreactivity for each antibody used in the study. Negative controls had the primary antibody omitted and replaced by Antibody Diluent (Dako, Glostrup, Denmark).

### Data analysis and statistical methods

Patients were subdivided into groups based on different clinical and pathological parameters, such as age, menopausal status, tumor size, nodal status, histological grade, desmoplastic reaction, tumor advancing ege, vascular invasion, perineural invasion, necrosis, mitotic index o peritumor inflammation. Differences in percentages were calculated with the chi-square test and Yates' correction when necessary. For quantitative comparison, levels of these biological parameters were expressed as a mean or a median (range). We analyzed the distribution of variables by the Kolmogorov-Smirnov test. On the base of this analysis, parametric methods (unpaired Student's and ANOVA tests) or non-parametric rank methods (Mann-Whitney and Kruskal-Wallis tests) were used for comparison between groups of patients. The SPSS 11.5 program (SPSS Inc, Chicago, IL, USA) was used for all calculations.

## Results

In overall, using ACR BI-RADS MR lexicon, a total of 19 cases were BI-RAD 4 and 49 cases were BI-RAD 5 III [16]. The MRI parameters obtained from the breast carcinomas included in the present study are shown in Table 1. Four cases showed a regional non-nodular enhancement, so the morphological features were only analyzed in 64 cases, whereas the kinetic features in all 68 cases. These parameters included: mass margins, internal mass enhancement, as well as kinetic characteristics such as time of peak of maximum enhancement, percentage of maximum enhancement before 2 min, and type of enhancement curve. As can be seen in the table, the majority of tumors showed an irregular or spiculated mass margin (75%), and a heterogeneous or rim internal mass enhancement (81,2%). Nevertheless, it is remarkable that homogeneous enhancement cases were only observed in T1 (<2 cm in size) tumors (28.6%), whereas none of the ≥ T2 tumors (≥ 2 cm) showed this finding (p = 0.015). Only 2 (3.1%) tumors showed rim enhancement. Likewise, the peak of maximum enhancement was more frequently shown by tumors after two min (75%), and a type III curve was shown by a higher percentage of cases (type I curve, 7.3%; type II, 35.3%; and type III, 57.4%). However, there was not a clear predominance in the percentage of maximum enhancement among tumors before two minutes.

**Figure 1:**
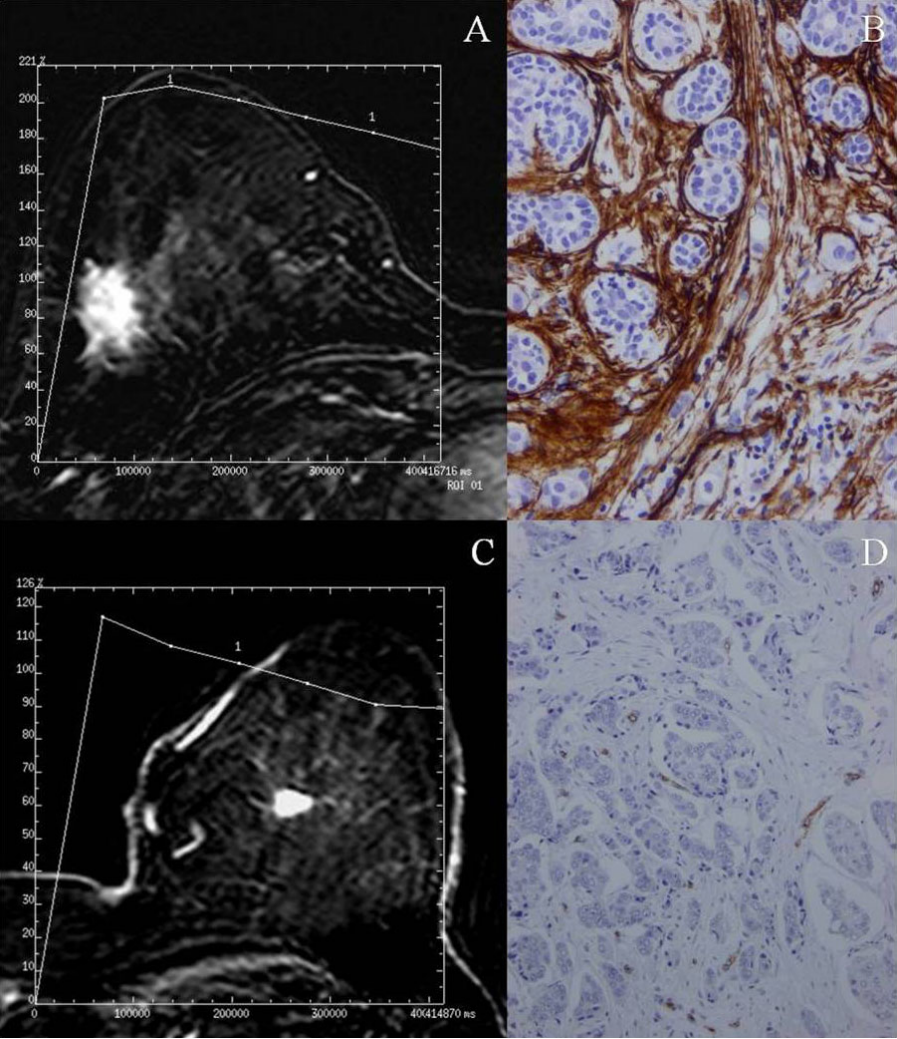
**Breast MR image acquired in a 50-years-old patient with a palpable mass in the right inferior outer quadrant**. The axial postcontrast subtracted image (9.9/4.2; flip angle, 10°) depicts a lesion with spiculated margin of mass (arrow). The time-signal intensity curve of this shows a type III time course with a peak of maximal enhancement after two min. (A). Immunohistochemical staining of CD34 in the same tumor showing a high microvessel density. 100×. (B). Breast MR image acquired in a 63-years-old patient with a palpable mass in the left upper inner quadrant. The axial postcontrast subtracted image (9.9/4.2; flip angle, 10°) depicts a lesion with smooth margin of mass (arrow). The time-signal intensity curve of this shows a type III time course with a peak of maximal enhance before 2 min (C). Immunohistochemical staining of CD34 in the same tumor showing a low microvessel density. 100×. (D).

Table 2 shows the relationship between morphological features, kinetic patterns of enhancement and the count of intratumoral microvessel density (MVD), by using immunostaining with anti-CD34. Tumors with irregular of spiculated mass margin had a significant higher MVD than tumors with smooth margin (p = 0.038). Likewise, we found that tumors showing a maximum enhancement peak at two minutes or longer, had a significant higher MVD count than those ones that reached this maximum point before two minutes (p = 0.012). Figure 1 shows representative examples of these associations.

**Table 2:** Relationship between morphological features, kinetic patterns of enhancement and the count of intratumor microvessel density (MVD)

RM features	N°	MVD Mean (range)	p
	
	64		
**Morphological features**			

Margin			**0.038**
Well defined	16	21.8 (4.9-54.4)	
Irregular/Spiculated	48	32.9 (5.2-140.6)	

Enhancement internal			n.s.
Homogeneous	12	19.5 (4.9-63.2)	
Heterogeneous/Rim	52	32.6 (5.2-140.6)	

**Kinetic features**	68		

Peak of maximum enhancement			**0.012**
< 2'	17	20.8 (8-54.4)	
≥ 2'	51	33.6 (4.9-140.6)	
	
% of maximun enhancement < 2'			n.s.
≤ 150%	39	31.2 (4.9-140.6)	
> 150%	29	29.3 (5.2-96.5)	
	
**Curve type**			n.s.
I	5	36.5 (16.3-60.5)	
II	24	29.6 (4.9-96.5)	
III	39	30.1 (8-140.6)	

* Four cases showed a non-nodular enhancement, so the morphological features were analyzed in only 64 cases, and the kinetic features in all 68 cases.

Abbreviations: MVD, microvessel density; n.s, not significant.

**Figure 2:**
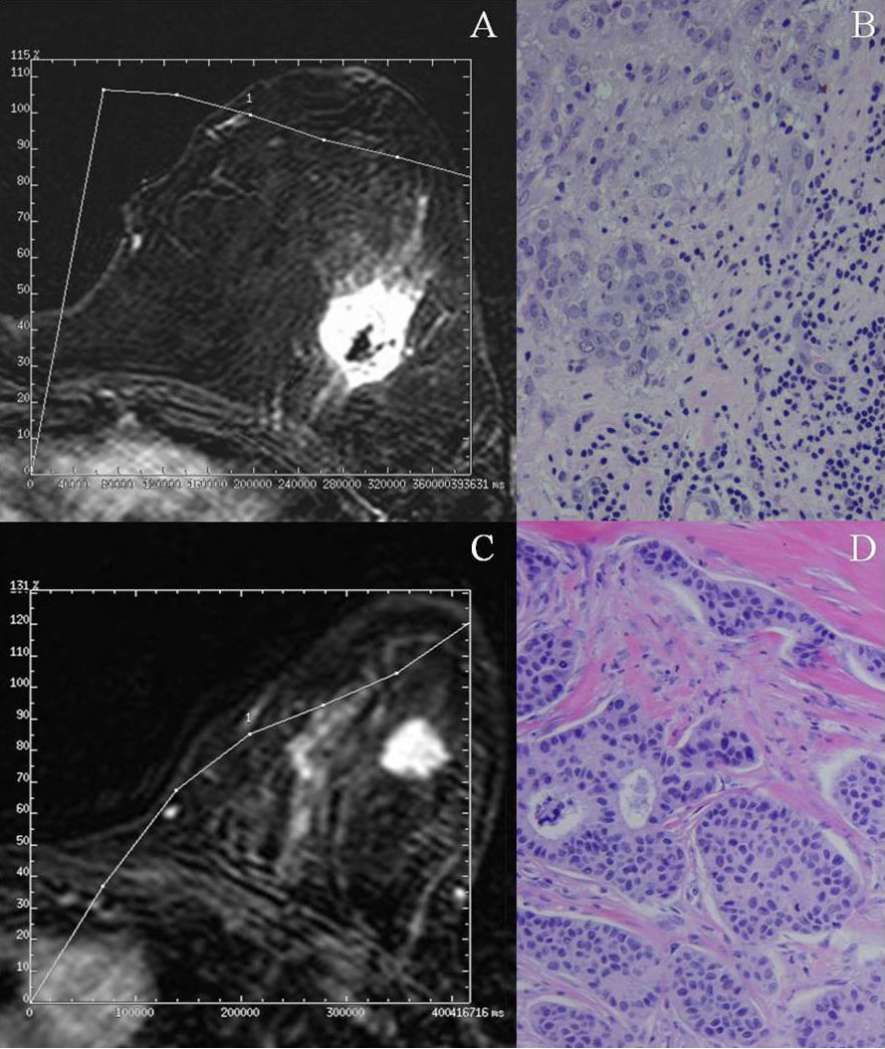
**Breast MR image acquired in a 45-years-old patient with a palpable mass in the left upper outer quadrant**. The axial postcontrast subtracted image (9.9/4.2; flip angle, 10°) depicts a lesion with irregular margin of mass and heterogeneous enhancement (arrow). The time-signal intensity curve of this shows a type III (washout) curve. **(A)**. Microphotography of the same tumor showing a peritumoral inflammation (arrows). 200×. **(B)**. Breast MR image acquired in a 63-years-old patient with a palpable mass in the left upper outer quadrant. The axial postcontrast subtracted image (9.9/4.2; flip angle, 10°) depicts a lesion of irregular margin of mass (arrow). The time-signal intensity curve of this shows a type I curve. **(C)**. Microphotography of the same tumor showing no a peritumoral inflammation. 100×. **(D)**.

Table 1 also shows the relationship between morphological features, kinetic pattern of enhancement and clinicopathological patient and tumor characteristics. Among all these factors, we found significant associations of MRI parameters with vascular invasion, mitotic index and peritumor inflammation. The percentage of tumors with vascular invasion or with high mitotic index was significantly superior among those showing a low percentage of maximum enhancement (≤ 150%) before two minutes than in those showing a high percentage (>150%) of enhancement rate at that time interval (p = 0.016 and p = 0.03, respectively). However, in contrast, there was a significant and positive association between the rate of mitotic index and the peak of maximum enhancement before two minuets (p = 0.036). Moreover, the percentage of tumors with peritumor inflammation was considerably higher in those showing type III curves than in those with either types I or II curves (p = 0.042), as well as when we compared both type I and type II with type III (p = 0.035). Figure 2 shows representative examples of this association. On the other hand, our results did not show significant associations between the different MRI parameters and menopausal status, nodal status, histotologial grade (Table 1), patient's age, desmoplastic reaction, tumor advancing edge, perineural invasion, necrosis, histological type, nuclear grade or DCIS associated component (data not shown).

In the present study we also analyzed the possible relationship between morphological features, kinetic patterns of enhancement and some biological parameters of interest in breast cancer, such as ER, PgR, p53, ki67, HER2, VEGFR-1 and 2. However, as Table 3 demonstrates, our results did not show any significant association between the MRI parameters and any of these biological factors.

**Table 3:** Relationship between morphological features, kinetics patterns of enhancement and biological parameters

Biological Parameters		Morphological features				N°	Kinetic features						
				
		Margin		Enhancement internal			Peak of maximum enhancement		% of maximum enhancement < 2'		Curve Type		
				
	N°*	A	B	Hom	Het/Rim		< 2'	≥ 2'	≤ 150%	> 150%	I	II	III
Total cases	64					68							

Estrogen receptors**													
Negative	13	3 (23.1)	10 (76.9)	2 (15.4)	11 (84.6)	14	3 (21.4)	11 (78.6)	9 (64.3)	5 (35.7)	-	9 (64.3)	5 (35.7)
Positive	51	13 (25.5)	38 (74.5)	10 (19.6)	41 (80.4)	54	14 (25.9)	40 (74.1)	30 (55.6)	24 (44.4)	5 (9.2)	15 (27.8)	34 (63)

Progesterone receptors**													
Negative	18	4 (22.2)	14 (77.8)	3 (16.7)	15 (83.3)	20	6 (30)	14 (70)	12 (60)	8 (40)	-	9 (45)	11 (55)
Positive	46	12 (26.1)	34 (73.9)	9 (19.6)	37 (80.4)	48	11 (22.9)	37 (77.1)	27 (56.3)	21 (43.7)	5 (10.4)	15 (31.3)	28 (58.3)

p53**													
Negative	52	11 (21.2)	41 (78.8)	9 (17.3)	43 (82.7)	56	14 (25)	42 (75)	31 (55.4)	25 (44.6)	5 (8.9)	19 (33.9)	32 (57.2)
Positive	12	5 (41.7)	7 (58.3)	3 (25)	9 (75)	12	3 (25)	9 (75)	8 (66.7)	4 (33.3)	-	5 (41.7)	7 (58.3)

HER2**													
Negative	38	10 (26.3)	28 (73.7)	8 (21.1)	30 (78.9)	41	9 (22)	32 (78)	23 (56.1)	18 (43.9)	5 (12.2)	15 (36.6)	21 (51.2)
Positive	26	6 (23.1)	20 (76.9)	4 (15.4)	22 (84.6)	27	8 (29.6)	19 (70.4)	16 (59.3)	11 (40.7)	-	9 (33.3)	18 (66.7)

Ki67**	64	17.5 (3-85)	20 (1-95)	17.5 (5-85)	20 (1-95)	68	20 (3-95)	15 (1-85)	20 (1-85)	15 (1-95)	5 (5-45)	15 (1-85)	20 (3-95)

VEGFR-1#	64	59.4 (0-216.1)	54.8 (0-191)	60.7 (0-216.1)	54.2 (0-191)	68	53.6 (0-216.1)	54.7 (0-191)	56.5 (0-216.1)	49.6 (0-139.5)	0 (0-103.4)	50.2 (0-145.8)	56.5 (0-216.1)

VEGFR-2#	64	40.9 (0-126.8)	37.7 (0-155.5)	18.8 (0-155.5)	39.7 (0-140.9)	68	39.2 (0-122.1)	34.1 (0-155.5)	36.2 (0-133.6)	32.8 (0-155.5)	0 (0-56.2)	34 (0-155.5)	39.2 (0-140.9)

* Four cases showed a non-nodular enhancement, so the morphological features were analyzed in only 64 cases, and the kinetic features in all 68 cases.

**Data are expressed as number of cases (percentage).

# Data are expressed as median (range).

Abbreviations: A, well-defined (regular); B, ill-defined (irregular o spiculated); Hom: homogeneous; Het: heterogeneous.

## Discussion

To evaluate the relationship between kinetic patterns and either clinical or biological variables, we chose the zones of maximum intensity of enhancement as areas of interest. We found significant associations of these MRI parameters with histopathological factors indicative of tumor aggressiveness, suggesting that the preoperative MRI may provide us with clinically useful information in invasive breast cancer.

It was of note that homogeneous enhancement was only observed in tumors smaller than 2 cm (T1), whereas none of the T2 tumors greater than 2 cm (≥ T2) showed this pattern of enhancement. This finding may be because larger size tumors (≥ T2) are biologically more heterogeneous lesions than T1 tumors.

Our results are in accordance with those of other authors that reported that classical prognostic factors, such as nodal status, tumor grading or hormone receptor status, do not correlate with MRI parameters in invasive breast cancer [17,18]. These findings seem to indicate that MRI could provide complementary prognostic to those provided by the classical factors. Likewise, our results did not show any significant associations between MRI findings and molecular parameters of interest in breast cancer, such as HER2, p53, ki67, and VEGFR-1 and 2. These latter findings could signify that MRI reflects a biological behaviour of breast carcinomas somehow not related to those molecular factors. Nevertheless, in the present study we have found significant and positive associations of MRI with several histopathological parameters of interest, such as MVD, peritumor inflammation, vascular invasion, and mitotic index. Some authors reported correlation of histological grade to enhancement pattern [10,19]. We also found higher kinetic features in grade I tumors than in grade III tumors, such as peak of maximum enhancement <2' of type III curve. However, the differences in these parameters did not achieve significant differences. We consider that this may be due to the small size of sample for this comparison as well as to the known inter-observer variation in histological grade evaluation [20,21].

It has been postulated that the rapid enhancement demonstrated by breast carcinomas after administration of contrast media is a direct result of tumor angiogenesis. MVD reflects the angiogenesis activity which constitutes a prerequisite for the growth of malignant tumors greater than 2 millimetres. MVD is considered a significant although weak prognostic factor in breast cancer. In the present study, we have found a variability of MVD among tumors, which appears to correspond to their biological heterogeneity. In addition, our data show that intratumor MVD was significantly associated with irregular mass margin as well as a maximum peak after 2 min. It seems reasonable that there is an association of high MVD with irregular margin since this morphology is associated with a more aggressive behaviour in breast cancer. However, the positive association between MVD and delay in the maximal peak of enhancement is a new finding. Some authors [22,23] have described a correlation of initial enhancement with MVD, but in more recent studies [24] this association could not be demonstrated. Nevertheless, it is important to consider that the divergent results may be a consequence of differences in the techniques of microvessel quantification as well as differences in the investigated tumor area. This latter aspect is important especially for a tumor showing heterogeneous enhancement. It is known that there may be discrepancies in MVD estimation when it is evaluated in the periphery or in the centre of the tumors. Thus, for example, it has been recently reported that the characteristic enhancement in the periphery of breast carcinomas at MRI is not caused by an elevated MVD in the tumor periphery but rather by a lower MVD in the tumor center [25]. We have analyzed the areas of higher MVD, by immunostining with anti-CD34, in the tumoral center, and these were correlated with the area that exhibits strongest enhancement on the first post-contrast image. Therefore, we consider that our finding may be because the highest MVD delayed the display of the maximum enhancement capacity of the tumors. This may be because the paramagnetic contrast spends more time in to fill in the very vascularized tumors. Thus, this date might contribute to the preoperative assessment of tumor angiogenesis, being potentially useful in selecting patients as candidates for new therapies based on the available anti-angiogenic strategies. Nevertheless, it is also known that tumor enhancement in MRI may be influenced by several factors, in addition to the extent and pattern of vascularization, such as vessel permeability, cellularity, interstitial pressure, and the fraction of the extracellular space [26]. As mentioned before, we have also analyzed the possible relationship between MRI parameters and the expression of both VEGFR-1 and 2 in breast carcinomas. These two tyrosine kinase receptors are the major mediators of the mitogenic and permeability-enhancing effects of vascular endothelial growth factor (VEGF) on cells. VEGF acts as a potent and selective endothelial mitogen, inducing a rapid and complete angiogenic response [27]. In addition, there are data suggesting that VEGF expression is associated with contrast diffusion [9]. However, our results did not show any significant associations between these growth-receptor expressions and MRI parameters.

Angiogenesis is necessary for a tumor to grow but not sufficient for it metastasize. There are biological differences between the ability of tumors to develop advanced neovascularization and their ability to invade blood vessels, this latter one being recognized as a more potent prognostic factor of poor outcome in breast cancer. In fact, in the present study we did not find any significant differences in MVD count between tumors with vascular invasion (mean MVD: 29.8 (range: 4.9-66.3)) and those without vascular invasion (30.9(5.2-146)). In this study, vascular invasion was defined as either the presence of neoplastic cells with fibrin clots, erythrocytes, or both within an endothelial-lined space without erythrocyte extravasation into the surrounding tissue or by the presence of neoplastic cells within a smooth muscle cell-lined space. Furthermore, we confirmed additional immunostaining with anti-CD34 as a pan-endothelial marker. Our results showed that the vascular invasion and a high mitotic index were significantly and positively associated with a low percentage of maximum enhancement (≤ 150%) before two minutes. These associations may be explained due a lower number of functional vessels because the occupation of these ones by cancerous cells with high proliferate rate, which prevent a fast passage of paramagnetic contrast. Even so, our data suggest a value for the dynamic MRI in assessing the metastatic potential of breast carcinomas. In contrast, there was a significant and positive association between the rate of mitotic index and the peak of maximum intensity before two minutes, which is in accordance with previous studies reporting a positive association between MRI parameters and other proliferative parameters, such as high DNA S-phase percentage [28] or proliferative cellular activity as shown by proliferative nuclear antigen (PCNA) immunoreactivity [22]. Therefore our data appears to indicate that the proliferative activity and the invasive potential of cancerous cells could be, at least partially, unrelated processes.

We also consider remarkable our finding of a significant relationship between peritumor inflammation and signal-intensity curves type III. Mononuclear inflammatory cells may account for as much as 50% of the total tumor mass in some invasive carcinomas. Historically, tumor-infiltrating leukocytes have been considered to be manifestations of an intrinsic defence mechanism against tumor development [29]. However, there are data indicating that leukocyte infiltration might promote tumor phenotypes, such as angiogenesis, growth, and invasion [30]. This may be because inflammatory cells probably influence cancer promotion by secreting cytokines, growth factors, chemokines and proteases, stimulating proliferation and invasiveness of cancerous cells [31]. This association between peritumor inflammation and a type III curve is, to the best of our knowledge, a new finding. A time course curve type III is a strong indicator of malignancy and is independent of other criteria [5]. In addition, the detection of a washout phenomenon suggests the presence of an increased vessel density and arterio-venous anastomoses with rapid outflow and thus fading of the contrast material [23]. Therefore, our finding may to contribute to the characterization of the possible biological influence of infiltrating mononuclear inflammatory cells in breast cancer.

Limitation of the present study is the difficult to asses exactly the tumor localization evaluated by MRI and histologically. Nevertheless, we consider that we performed a reasonable correlation between both types of evaluations. Certainly there are other possible parameters for evaluating MRI and both histological and biological aspects of breast carcinomas, which should be investigated in futures studies. In addition, prospective studies are necessary to asses the potential value of MRI parametrers as prognostic factors in breast cancer.

## Conclusions

In conclusion, especially relevant are our findings that variations in the dynamic MRI parameters seem to be associated with parameters indicatives of tumor aggressiveness, such as high MVD count, vascular invasion, high mitotic index in breast cancer or peritumor inflammation. Therefore, our results are in accordance with previous report indicating the potential value of dynamic MRI for better characterizing breast cancer.

## Competing interests

The authors declare that they have no competing interests.

## Authors' contributions

guarantor of integrity of the entire study- FGO; study concepts and design- FGO, LH, LMA and VF; literature research- FGO, LH and VF; clinical studies- FGO, GPP and VF; experimental studies/data analysis-FGO, GL, GRS, AA and; statistical análisis- A and; manuscript preparation- FGO and VF; manuscript editing- FGO and GRS. All authors read and approved the final manuscript.

## Pre-publication history

The pre-publication history for this paper can be accessed here:

http://www.biomedcentral.com/1471-2407/10/8/prepub
